# Decoding pre-movement neural activity from thalamic LFPs for adaptive neurostimulation in tremor patients

**DOI:** 10.1016/j.neucom.2026.132899

**Published:** 2026-04-14

**Authors:** Fernando U. Rodriguez Plazas, Thomas G. Simpson, Laura Wehmeyer, Rahul S. Shah, Jamie Brannigan, Michael G. Hart, Pablo Andrade, Francesca Morgante, Veerle Visser-Vandewalle, Erlick A. Pereira, Huiling Tan, Shenghong He

**Affiliations:** a Medical Research Council Brain Network Dynamics Unit, Nuffield Department of Clinical Neurosciences, University of Oxford, Oxford, UK; b Medical Research Council Centre of Research Excellence in Restorative Neural Dynamics, UK.; c Neuromodulation and Motor Control Section, Neuroscience and Cell Biology Research Institute, City St George’s, University of London, London, UK; d Nuffield Department of Surgical Sciences, University of Oxford, Oxford OX3 9DU, UK; e Department of Stereotactic and Functional Neurosurgery, Faculty of Medicine and University Hospital Cologne, University of Cologne, Cologne, Germany

**Keywords:** Movement decoding, Local field potentials, Neural decoding, Electroencephalography, Ventral intermediate nucleus, Thalamus, Tremor disorders, Machine learning, Deep brain stimulation

## Abstract

**Objective:**

To advance adaptive deep brain stimulation for tremor disorders, we investigated the feasibility of using machine learning to decode pre-movement oscillatory changes in thalamic local field potentials (LFPs) and scalp electroencephalography (EEG) signals. Our aim was to predict upcoming upper-limb movements based on these neural signals.

**Approach:**

We recorded and analysed from 11 patients undergoing deep brain stimulation surgery for the treatment of tremor, employing machine learning models—including logistic regression, gradient-boosted decision trees, and convolutional neural networks—to distinguish rest periods from pre-movement periods.

**Main results:**

We demonstrate that early neural correlates can predict movement onset, achieving above-chance decoding performance starting approximately 430 ms before movement initiation using thalamic LFP and 840 ms using EEG signals. Individualised, patient-specific decoders outperformed cross-patient models, reflecting inter-patient variability in neural modulatory patterns. Additionally, multiple frequency bands contributed independently to decoding performance, highlighting the importance of incorporating a spectrum of frequencies rather than relying solely on activity in any single canonical band.

**Significance:**

These findings underscore the value of personalised, multi-band machine learning-based approaches for capturing the neural correlates preceding movement. They support the development of adaptive neurostimulation therapies through tailored models that account for patient-specific patterns in neural activity.

## 1 Introduction

Since receiving FDA approval for the treatment of essential tremor (ET) in 1997, deep brain stimulation (DBS) of the ventral intermediate nucleus (VIM) of the thalamus has established itself as an effective therapy for tremor disorders in patients with medication-refractory symptoms [1], [2], [3]. In a meta-analysis comprising 1714 ET patients, VIM-DBS improved tremor scores by a mean 61.3 % at 20 months follow-up, showing a significant therapeutic effect for tremor suppression [4].

Despite its efficacy for managing tremor, VIM-DBS is associated with several adverse effects, including stimulation-induced side-effects that impact speech and postural stability, as well as a gradual loss of therapeutic efficacy over time [5], [6], [7], [8], [9], [10], [11], [12]. To address these challenges, non-continuous or adaptive DBS (aDBS) schemes have been proposed as a means to mitigate the side effects associated with stimulation and thereby increase DBS’s therapeutic window [13], [14], [15], [16].

A critical aspect in the development of aDBS systems is the selection of appropriate feedback signals to guide the titration of stimulation. Various surrogate signals have been explored for driving aDBS in ET patients, including muscle activity recorded from surface electromyography [15], [17], [18], scalp electroencephalography (EEG) [19], and signals recorded from intracranial electrodes [14], [20], [21]. However, there remains an interest in leveraging the modulatory patterns found in neural signals directly recorded from the implanted DBS electrodes themselves to avoid the need for supplementary implants or external devices [22]. By capturing the neural correlates of movement through local field potentials (LFPs) measured from the thalamus, it is possible to detect movement states and utilise this information to drive an aDBS system [23]. Prior studies have successfully implemented this aDBS approach in an acute clinical setting [24], [21], [25], [26]

In the context of aDBS for patients with intention tremors (tremors that emerge during voluntary movements rather than at rest), the timing of stimulation is critically important. Early triggering of stimulation—ideally *prior to or immediately at* the onset of voluntary movement—holds strong therapeutic potential. Clinical and experimental observations [14], [15] show that even with responsive DBS that switches on when tremor begins, patients typically experience a brief burst of tremor just after movement onset – before stimulation has had time to achieve its therapeutic effect. For instance, Opri et al. [14] reported that, in their cohort, aDBS triggered by movement onset still resulted in a short, visible burst of tremor. By initiating stimulation based on pre-movement (e.g., thalamic LFPs or cortical potentials) as opposed to movement-related signatures, one can potentially disrupt tremor-related oscillations before they fully develop. In contrast, reactive approaches that wait for tremor to appear before delivering stimulation cannot prevent this initial burst and may therefore offer less complete symptom relief. This pre-emptive strategy—intercepting neural processes that give rise to tremor—could reduce or even eliminate tremor episodes before they begin.

Achieving this, however, requires systems capable of decoding movement onsets in real time, for instance by detecting neural correlates present in the period leading up to movement initiation. Such early detection of movement from neural signals has already been demonstrated, and electrocorticography (ECoG) signals have been shown to yield higher decoding performance than LFP signals recorded from the subthalamic nucleus of Parkinson's disease (PD) patients [27]. However, whether thalamic LFPs carry equally discriminable pre-movement patterns in tremor patients remains unknown.

In this study, we hypothesise that it is possible to train machine learning models to decode pre-movement neural signatures of volitional upper-limb motor activity from both thalamic LFPs and scalp EEG with above-chance accuracy at least several hundred milliseconds before movement onset. To test this, we develop and implement multiple machine learning-based classification pipelines—ranging from feature-based logistic regression and gradient-boosted trees to convolutional neural networks that learn directly from raw oscillatory inputs. We compare the models’ decoding performance, determine how early relative to movement onset oscillatory signatures can be detected, and study the oscillatory features that drive the models’ predictions.

The remainder of this manuscript is structured as follows: we first introduce the dataset, feature extraction pipelines, and classification algorithms used in this study. We then present the decoding performance achieved by these systems and document the neural correlates that they rely on to make inferences. Finally, we discuss the trade-offs of the different approaches and their implications for early movement-driven aDBS in tremor disorders.

## 2 Methods

### 2.1 Participants and experimental protocol

Data were collected from 11 patients (5 female) undergoing DBS surgery for tremor management. The cohort included nine patients with ET, one with Orthostatic Tremor (OT), and one with tremor-dominant Parkinson’s disease. Clinical information for each participant is provided in Table 1. All procedures were conducted in accordance with the Declaration of Helsinki, with informed written consent obtained from all participants prior to their inclusion in the study; and approved by the relevant local ethics committees (SGH: MED IDREC Ref 18/SC/0436, IRAS 249989; UHC: Ethics Committee of the Medical Faculty of the University of Cologne No. 20–1054).

**Table 1:** Clinical and demographic patient information.

**Patient ID**	**Diagnosis**	**Centre**	**DBS System**	**Surgical Target**	**Sex**	**Age [years]**	**Disease Duration [years]**	**Predominant Symptoms Before Surgery**
S1	ET	SGH	Abb	VIM	F	77	21	Tremor, gait ataxia, tremor worse on right, upper limb and voice tremor
S2	ET	SGH	Abb	VIM	M	70	8	Tremor, upper limb, with right worse than left, lower limb tremor
S3	ET	SGH	Abb	VIM	F	62	45	Tremor, upper limb tremor left worse than right, voice tremor
S4	ET	SGH	Abb	VIM	M	70	5	Tremor, upper limb left worse than right
S5	ET	UHC	Med	VIM/PSA	F	58	15	Tremor in both hands, left hand worse than right
S6	ET	UHC	Med	VIM/PSA	M	72	10	Tremor in both hands (stronger in right hand), some head tremor
S7	ET	SGH	Med	VIM/ZI	M	64	20	Tremor
S8	ET	SGH	Med	VIM/ZI	M	71	16	Tremor
S9	ET	SGH	Med	VIM/PSA	F	59	50 +	Tremor
S10	PD	UHC	Med	VIM/PSA	M	68	6	Rigidity and tremor in right hand and arm
S11	OT	UHC	Med	VIM/PSA	F	61	9	Orthostatic and action tremor in both hands

Abbreviations: ET = Essential Tremor; PD = Parkinson’s disease; OT = Orthostatic Tremor; SGH = St. George’s Hospital; UHC = University Hospital Cologne; Abb = Abbott Infinity DBS System; Med = Medtronic SenSight DBS System.

Patients underwent stereotactic neurosurgery for bilateral implantation of DBS leads targeting the VIM, Zona Incerta (ZI), or posterior subthalamic area (PSA) for the treatment of tremor. For research purposes, the DBS leads were temporarily externalised for up to seven days, allowing direct recording of neural signals before connecting them to the implantable pulse generator (IPG).

Four patients (S1–S4) were implanted with the Abbott Infinity DBS system (Abbott Laboratories, US), which uses eight-contact directional leads arranged in a 1–3-3–1 layout (two ring contacts at the ends and two central levels each split into three segments). The remaining seven patients (S5–S11) were implanted with Medtronic SenSight directional leads, also arranged in a 1–3–3–1 configuration (eight contacts per lead). LFPs were recorded in unipolar mode with either a TMSi Porti amplifier (TMSi, The Netherlands) at 2048 Hz and 22-bit resolution, or a TMSi SAGA amplifier at 4096 Hz and 24-bit resolution. An on-board digital *sinc*³ anti-aliasing filter (cut-off frequency of 553 Hz and 1.6k Hz, respectively) was automatically applied to all recorded signals. A wrist-worn electrode was used as a temporary hardware reference during data collection; however, none of our analyses relied on that wrist reference directly. Instead, to eliminate any residual common-mode or motion-related artifacts (including potential contamination from wrist movement or muscle activity), all LFP channels were re-referenced online to the common average of all simultaneously recorded LFP channels. This common-average re-referencing ensured that any voltage fluctuations specific to the wrist electrode (e.g. motion) were effectively subtracted from the recorded signals prior to further processing.

Scalp EEG was recorded simultaneously using electrodes at Cz, C3, C4, CPz, CP3, and CP4 (10–20 system), covering somatosensory, sensorimotor, and motor areas. EEG signals were captured on the same amplifiers (with identical on-board filtering) and sampled synchronously with the LFPs. Surface EMG was recorded with bipolar electrodes over the forearm flexor and extensor muscles; these EMG traces provided precise markers of upper-limb movement onset for temporal alignment with the neural data.

During recording sessions, patients were instructed to perform a series of upper limb motor tasks designed to elicit voluntary movement: a rice pouring task (patients poured rice back and forth between two cups for approximately 10 s before returning to rest), pegboard insertion task (patients inserted pegs into a pegboard for approximately 10 s before returning to rest), and foam ball gripping task (patients tightly gripped a foam ball with one hand for approximately 5 s before returning to rest). Each task was performed multiple times with rest intervals to prevent fatigue. Instructions were standardised, and patients were encouraged to perform movements at a comfortable pace, with plenty of rest (at least 10 s between trials) in between. In total, a mean of 67.18 ± 38.8 trials (cross-patient mean ± std. deviation; min: 16, max: 112) were used for analysis.

All signals—including thalamic LFPs, scalp EEG, and surface EMGs—were recorded using custom-developed software tailored for electrophysiological data acquisition. Recorded data were digitised and stored securely for offline processing and analysis.

### 2.2 Data pre-processing and labelling

EMG signals were recorded from bipolar electrodes placed over the forearm flexor and extensor muscles to determine the timing of movement initiation. For lateralised tasks (e.g., one-handed gripping), only the EMG trace from the active arm was used. For bilateral tasks (pegboard insertion, rice pouring), each arm’s EMG was processed independently, and the earliest detected onset time—regardless of which arm moved first—was taken as the movement initiation time.

Raw EMG traces were first high-pass filtered using a causal fourth-order Butterworth filter implemented in a cascade of second-order sections with a cut-off frequency of 0.5 Hz. To minimise ringing artifacts due to filter warm-up, the initial filter state was adjusted, and all filtering was performed in a forward-only (causal) manner to preserve the temporal integrity of the signals and mimic signal processing techniques that could be applied during real-time use [28]. Following high-pass filtering, the magnitude of the EMG signals was computed to obtain an envelope of muscle activity, and z-scored to standardise the amplitude across trials and participants. These processed EMG traces were visually inspected to ensure clear delineation of movement initiation, and traces that did not distinctly define and isolate movement onset were discarded. This signal was then rectified and smoothed, and a threshold (three times the standard deviation extracted from the baseline period) detected the first point at which deviation from baseline activity was detected (i.e. movement initiation). These timings were subsequently reviewed manually to ensure movement initiation was set at the earliest possible time point. This manual labelling of the movement onset served as the ground truth for subsequent analyses.

To prevent the introduction of unwanted phase lags between the movement signals and the neural signals, identical high-pass filtering (causal fourth-order Butterworth filter with a cut-off frequency at 0.5 Hz) was applied to the thalamic LFP and cortical EEG signals [28]. This approach ensured temporal alignment across different kinds of signals. Additionally, to eliminate power line interference and harmonics, notch filters were applied at 50 Hz, 100 Hz, and 150 Hz using a causal filter design implemented in cascaded second-order sections.

The movement onset times defined from the EMG signals were used to segment the LFP and EEG data into epochs corresponding to individual movement trials. For participants who performed multiple movement-related tasks (gripping, rice pouring, and pegboard insertion), the epochs from all tasks were combined for the decoding analysis.

### 2.3 Time-resolved characterization of oscillatory activity

**Fig. 1:**
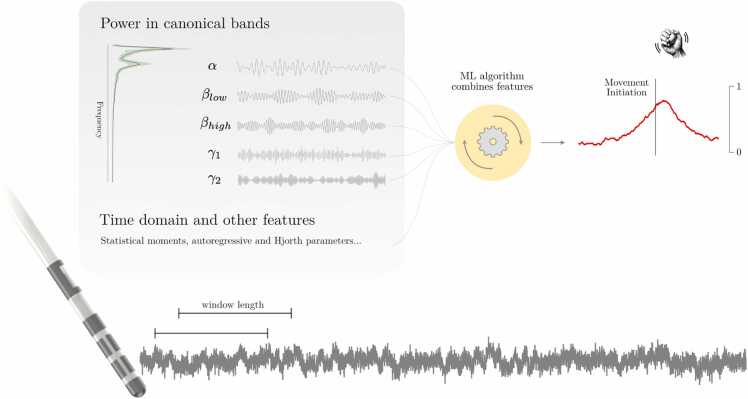
Feature-Extraction Pipeline for Real-Time Pre-Movement Decoding. Raw LFP and EEG signals are first pre-processed (high-pass and notch filtering) and then segmented into two overlapping trailing windows (1 s and 2 s), updated every 20 ms. Within each window, we compute spectral, time-domain, autoregressive, and cepstral features. Each feature set is normalised using causal running (10 s) z-scores, then concatenated into a single feature vector. These vectors—computed entirely from past data at each 20 ms step—serve as inputs to classifiers that detect pre-movement periods in real time.

To train decoders that detect pre-movement periods, we implemented a feature-extraction pipeline (Fig. 1) that captures time-resolved oscillatory activity. Time series signals were first segmented into overlapping trailing windows (1 s and 2 s), from which features were extracted and then concatenated. By providing both window lengths as separate inputs, the classifier can learn which timescale best captures pre-movement signatures. Feature vectors were produced every 20 ms (i.e. a 50 Hz update rate).

To maintain full causality—that is, to ensure no future samples influence current estimates—each window ends at the current time point and only uses data from the past. Immediately after extracting each feature vector, we standardised it using a running (causal) z-score: for each feature dimension, we subtract the mean and divide by the standard deviation computed over the previous 10 s of data. This trailing window ensures that, at time *t*, all statistics (mean and *σ*) are based solely on samples up to *t*, preventing any leakage of future information [29].

At each time point, this procedure yields a normalised vector comprising several features that reflects oscillatory dynamics.

### 2.4 Power in canonical frequency bands

For each time window of electrophysiological data, we first applied a Hann taper w[n] to the time-domain signal x[n] to reduce edge artifacts and then computed its DFT $X\left[k\right]$. The log-power spectrum is given by

$$L\left[k\right]=\log\left({\left|X\left[k\right]\right|}^{2}\right)$$

From L[k] we extracted, for each canonical band *B*, the mean log-power $\mu_{B}=\frac{1}{\left|B\right|}\sum_{k\in B}L\left[k\right]$ in bands theta (4–8 Hz), alpha (8–12 Hz), low beta (13–20 Hz), high beta (20–30 Hz), gamma (30–60 Hz, 60–80 Hz, 80–100 Hz), and high-frequency activity (100 – 200 Hz, 200–500 Hz).

### 2.5 Time-domain statistics

We extracted time-domain statistics to capture the characteristics of the signal directly from its temporal representation. The primary time-domain features included the first four statistical moments of the signal distribution, as well as the Hjorth parameters.

The first four statistical moments are:

• **Mean (First Moment):** Average value of the signal over time. Provides a measure of the central tendency, the overall level of activity within the signal, as well as a measure of low-frequency activity. $\mu_{x}=\frac{1}{N}\sum_{n=0}^{N-1}x\left[n\right].$
• **Variance (Second Moment):** Computed as a Hjorth parameter (*Activity*, see below). $\sigma_{x}^{2}=\frac{1}{N}\sum_{n=0}^{N-1}{\left(x\left[n\right]-\mu_{x}\right)}^{2}.$
• **Skewness (Third Moment):** Quantifies the asymmetry of the signal's amplitude distribution, detecting deviations from normality in the signal's amplitude. $\text{Skewness}=\frac{1}{N}\sum_{n=0}^{N-1}{\left(\frac{x\left[n\right]-\mu_{x}}{\sigma_{x}}\right)}^{3}$.
• **Kurtosis (Fourth Moment):** Describes the "tailedness" or “peakedness” of the signal distribution. High kurtosis indicates the presence of infrequent, significant deviations from the mean, potentially associated with transients or bursts in the signal. $\text{Kurtosis}=\frac{1}{N}\sum_{n=0}^{N-1}{\left(\frac{x\left[n\right]-\mu_{x}}{\sigma_{x}}\right)}^{4}.$

The Hjorth parameters are a set of three descriptors useful in the analysis of non-stationary signals, such as LFP, EEG, or MEG data, and provide a compact representation of the signal's dynamic properties:

• **Activity:** Corresponds to the variance of the signal, representing the overall signal power / energy content. $\text{Activity}=\sigma_{x}^{2}$
• **Mobility:** Defined as the square root of the variance of the first derivative of the signal divided by the variance of the signal itself. It quantifies the mean frequency or the rate of change in the signal, with higher mobility indicating faster signal fluctuations. $\text{Mobility}=\sqrt{\frac{\mathrm{Var}\left(\Delta x\right)}{\mathrm{Var}\left(x\right)}}$.
• **Complexity:** Measures the signal's waveform complexity relative to a pure sine wave, calculated as the ratio of the mobility of the first derivative of the signal to the mobility of the signal itself. It reflects the degree of variability in the frequency content of the signal, with higher values indicating more complex, non-sinusoidal waveforms. $Complexity=\sqrt{\frac{\mathrm{var}\left(\Delta^{2}x\right)}{\Delta x}}/\sqrt{\frac{\mathrm{var}\left(\Delta x\right)}{\mathrm{var}\left(x\right)}}$,

where$\Delta x\left[n\right]=x\left[n\right]-x\left[n-1\right],$and $\Delta^{2}x\left[n\right]=\Delta x\left[n\right]-\Delta x\left[n-1\right].$ Together, these time-domain features provided a comprehensive characterization of the electrophysiological signals, capturing essential aspects of the signal's amplitude, variability, and temporal structure. They are commonly used in various signal processing applications, including brain-computer interfaces, cognitive state monitoring, and the identification of pathological activity such as epileptic seizures [30], [31], [32].

### 2.6 Other features

We also extracted autoregressive (AR) parameters and cepstral coefficients to capture additional signal dynamics.

For the **autoregressive parameters**, we employed AR modelling to describe a signal as a linear combination of its 10 previous values, estimated by solving the Yule-Walker equations, which relate the autocorrelation function of the signal to the AR coefficients.

The **cepstral coefficients** were derived by taking the inverse Fourier transform of the logarithm of the signal's frequency spectrum. This technique can reveal periodic structures in the frequency domain. The cepstrum was segmented into 10 bands, and the average power within these bands computed.

### 2.7 Classification pipeline and evaluation of decoding performance

**Fig. 2:**
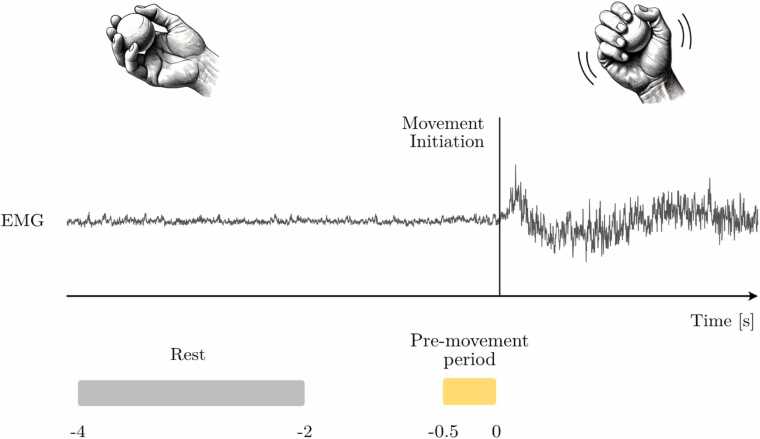
Illustration of the trial labelling process. Movement initiation is determined based on the earliest point at which EMG activity deviates from baseline.

To train classifiers capable of detecting pre-movement periods, as shown in Fig. 2, we labelled time windows ending within 500 ms prior to movement initiation as belonging to the *pre-movement period*. Windows ending within the interval [-4, −2] seconds relative to movement initiation were labelled as the *rest period*. Our classifiers were trained to distinguish between these two periods based on the features extracted from the corresponding electrophysiological timeseries.

The performance of the classifiers was evaluated using a leave-one-trial-out cross-validation scheme (LOTO-CV). In each iteration, one trial was reserved as validation data (which remains unseen by the classifier during training) to benchmark the classifier's out-of-sample performance, while the remaining trials were used to train the classifier—setting its parameter values and weights. This process was repeated iteratively, with each trial serving as the validation set once and as part of the training data in all other iterations. This approach ensures that all trials are used once for out-of-sample testing, providing an estimate of the classifier's generalization performance.

During each iteration, we computed the area under the Receiver Operating Characteristic curve (ROC-AUC; abbreviated AUC) for the predictions made by the trained classifier on the validation data. This metric was then averaged across all of the cross-validation folds to obtain a single, cross-validated performance score. To account for class imbalances—specifically, the longer duration of the rest period compared to the pre-movement period—we adjusted the ROC curve by differentially weighting samples based on the period from which they were extracted. This weighting corrected for the larger number of samples in the rest than in the pre-movement period. We performed this procedure for each EEG/LFP channel individually, which provided a decoding performance score (AUC) for every channel. Then, the best-performing channel from each hemisphere was chosen, and their features concatenated into a single feature vector to train an individual classifier. For EEG signals, the two best-performing channels were chosen. This schema provided a single decoding performance datapoint for each patient, driven by information from the most informative channel from each hemisphere–following a data-driven approach that dynamically selects channels based on their contributions to decoding performance.

We implemented and benchmarked four following classification architectures: logistic regression, gradient-boosted decision trees, convolutional neural network, and convolutional neural network with manually extracted features.

### 2.8 Logistic regression

Logistic Regression (LR) is a simple yet powerful classification algorithm that models the probability of a binary outcome using a linear combination of input features. The logistic regression model computes a linear combination of the features and applies the logistic (sigmoid) function to produce an estimate of the probability that the input features belong to the positive class $p\left(y=1\;\right|\mathrm{features})$.

In our implementation, during each iteration of the leave-one-trial-out cross-validation, we performed an internal three-fold cross-validation on the training data to determine the optimal level of regularization (i.e. the L2 penalty term). During training, sample weights were adjusted to correct for class imbalance by giving more weight to samples from the minority class. Using these selected hyperparameters, a final logistic regression model was trained on the training data and evaluated on the validation trial for the current fold. We used Python’s *scikit-learn* implementation of LR [33].

### 2.9 Gradient-boosted decision trees

Gradient-Boosted Decision Trees (GBDT) is an ensemble learning method that builds a predictive model by sequentially combining multiple decision trees learners, with each new tree aiming to correct the errors of the preceding ensemble. This iterative process is guided by gradient descent optimization, minimizing the negative log-likelihood.

In our implementation, we tuned the GBDT model to find the optimal hyperparameters, including the number of trees, learning rate, and tree depth, through an internal cross-validation procedure at each iteration of the leave-one-trial-out scheme. We utilised a grid search to explore a range of hyperparameter values, selecting the combination that yielded the best cross-validated performance on the training split of the data. Once the optimal hyperparameters were determined, the GBDT model was trained on the entire training set of the current fold and evaluated on the validation trial. Sample weighting was applied during training to account for dataset imbalance.

Our primary motivation for benchmarking GBDTs alongside logistic regression was to assess the potential benefits of capturing non-linear relationships between features and labels. If significant non-linear relationships existed in the data, GBDTs were expected to better capture and leverage these patterns compared to the linear logistic regression model. We used the Python *LightGBM* implementation of GBDTs [34].

### 2.10 Convolutional neural network

Convolutional neural networks (CNN) are deep learning models that automatically learn hierarchical feature representations from raw input data through multiple layers of convolutional filters. Unlike the feature-based methods described above, CNNs take minimally pre-processed electrophysiological time series as input, without relying on manually extracted features [35].

**Fig. 3:**
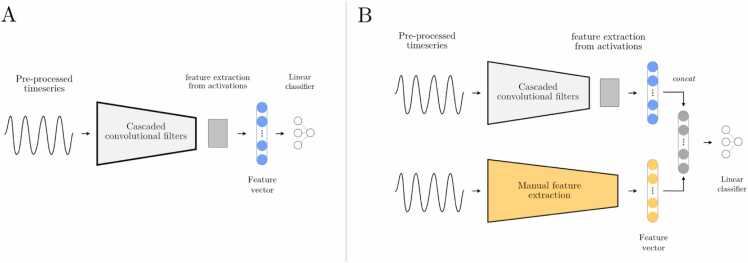
Convolutional neural network architectures CNN and FeatCNN. For the CNN (Panel A), pre-processed electrophysiological time series signals are processed through a cascade of convolutional filters. Activations are filtered, scaled, combined, and passed through non-linear activation functions as they progress through the layers of the network. Final activations are summarised using descriptive statistics, which serve as input features for a linear classifier, producing the final logit. The FeatCNN combines manually extracted features into the feature vector (Panel B).

In our CNN architecture (Fig. 3A), the input time series were processed through a cascade of 6 convolutional layers interleaved with Swish non-linear activation functions [36], batch normalization layers (to correct for scaling differences and improve training stability), and pooling layers to reduce the dimensionality of activations as the signals flow through the network. The convolutional filters are trained to extract relevant information content from the signals pertinent to the classification task. The training process involved the use of adaptive moment estimation (Adam), an adaptive gradient descent algorithm that iteratively adjusted the filter coefficients to minimise the cross-entropy loss between the model's predictions and the true labels.

At each stage of the network, the processed signals (referred to as "activations") were filtered, scaled, combined, and passed through non-linear activation functions. After the final convolutional layer, descriptive statistics were computed from the activations, producing scalar features that were input into a fully connected linear classification layer that yielded the final output logits.

### 2.11 Convolutional neural network with manually extracted features (FeatCNN)

To combine the strengths of both learned and hand-crafted representations, we designed a hybrid model (FeatCNN) that retains the same convolutional backbone as our pure CNN but also incorporates manually extracted features. In FeatCNN, the time-series input was first passed through the six-layer convolutional network—each convolutional filter trained end-to-end from random initialization, just as in the standalone CNN. Once the convolutional layers generate their activation outputs, these learned features were concatenated with the handcrafted feature vector (which includes band-power measures, Hjorth parameters, autoregressive coefficients, and cepstral coefficients). Handcrafted features were computed prior to downsampling the signals to 512 Hz. The merged feature set was then fed into the final fully connected layers for classification (Fig. 3B).

Because FeatCNN has access to both learned and handcrafted features, backpropagation drives its convolutional filters to capture patterns that complement—rather than simply duplicate—the information provided by the handcrafted features. In contrast, the pure CNN must discover relevant discriminative patterns directly from the time series, without handcrafted guidance. Training both architectures from scratch (i.e., with random weight initialization) ensured a fair comparison: in the CNN, filters must learn every useful signal characteristic, whereas in FeatCNN, the convolutional filters learn only those residual or orthogonal features that are not already encoded in the handcrafted set. By evaluating these two models, we were able to determine whether supplying handcrafted features alongside learned ones improved decoding.

For both convolutional network architectures, the electrophysiological time series signals were downsampled to 512 Hz from the original sampling rates (2048 Hz or 4096 Hz) before being passed to the network. We trained each CNN in a leave-one-trial-out cross-validation (LOTO-CV) framework: in each fold, all windows from a single movement trial were held out as the test set, and the remaining trials formed the training set. Within each training set, we applied manifold mixup augmentation in the logit space to encourage smoother decision boundaries and improve generalization [37]. To correct for the class imbalance between the relatively short pre-movement windows and the longer rest windows, we used weighted sampling so that pre-movement samples were oversampled during training. Each network was trained for a fixed 150 epochs per LOTO fold without early stopping. Neural network architectures were implemented in Python using *PyTorch* [38].

## 3 Results

### 3.1 Decoding of pre-movement periods

We evaluated the performance of four classification models—LR, GBDT, CNN, and FeatCNN—in distinguishing rest periods from pre-movement periods using thalamic LFPs and scalp EEG signals.

**Fig. 4:**
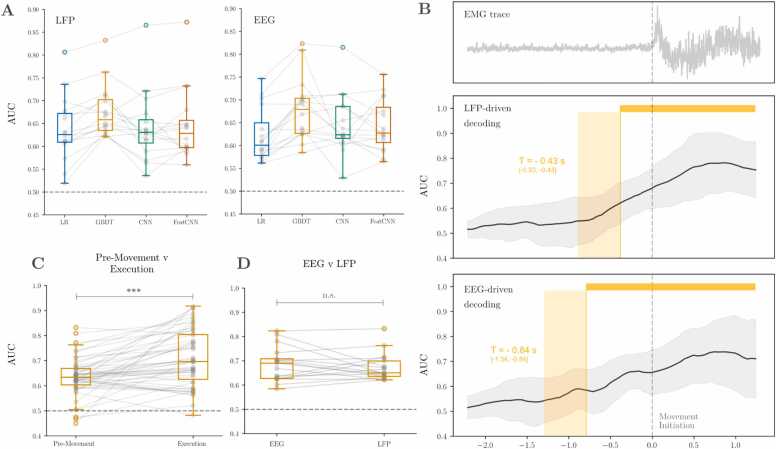
Decoding performance of different models for detecting pre-movement periods based neural oscillatory data. (A) Model performance: area under the receiver operating characteristic curve (AUC) values for each of four classification model for distinguishing rest from pre-movement periods using LFP and EEG data. The boxplots represent the distribution of AUC values across participants; individual dots outside the whiskers denote patients that are outliers. (B) Time-resolved decoding performance: Plots showing the time-resolved AUC values for logistic regression models trained to detect pre-movement periods at various time windows relative to movement initiation (t = 0 s; vertical dashed line). The top subplot displays the cross-participant average EMG trace, indicating the timing of muscle activation. The middle (LFP data) and bottom (EEG data) subplots illustrate the progression of cross-patient AUC values over time (solid black lines represent the mean AUC, and the grey shaded areas indicate ±1 cross-patient standard deviation). The shaded yellow region indicates the 500 ms region during which decoding performance reaches significant above chance level (AUC = 0.5), and the vertical yellow line indicates the end of this period, which is taken as the time point at which above-chance performance is achieved (C) Pre-movement decoding vs. decoding of movement execution: comparison of AUC values for GBDT models trained to detect pre-movement periods (from –500 ms to 0 ms relative to movement onset) versus movement execution periods (0 ms to 500 ms) using LFP data. All LFP channels are compared individually. This comparison assesses the model’s effectiveness for decoding neural activity immediately before movement initiation versus during movement execution. (D) Comparison between signal modalities: AUC values for GBDT models utilizing LFP and EEG data, illustrating the difference in decoding performance between the two neural signal modalities, highlighting the relative effectiveness of invasive (LFP) versus non-invasive (EEG) recordings in detecting pre-movement neural states.

Fig. 4A illustrates the area under the Receiver Operating Characteristic curve (AUC) values for each model using LFP and EEG data. Higher AUC values indicate better decoding performance. Mean AUC values and the corresponding 95 % confidence intervals are presented in Table 2.

**Table 2:** Mean AUC value [95% CI] for decoding the [-500 ms, 0] pre-movement period with different models. Higher values indicate better decoding performance.

	LR	GBDT	CNN	FeatCNN
LFP	0.64 [0.60, 0.67]	**0.68 [0.65, 0.70]**	0.64 [0.60, 0.68]	0.65 [0.61, 0.68]
EEG	0.62 [0.59, 0.65]	**0.68 [0.65, 0.71]**	0.65 [0.61, 0.68]	0.64 [0.62, 0.67]

Paired *t*-tests were conducted to compare model performance (AUC) across patients. Normality of the paired differences was assessed with the Shapiro-Wilk test; no strong deviations were detected (all *p*_*shapiro*_ > 0.05). *p*-values were corrected for multiple comparisons using the False Discovery Rate (FDR) procedure. For the LFP-driven models, GBDTs significantly outperformed the other models. Specifically, the comparison between LR and GBDT (*p* = 0.002), between CNN and GBDT (*p* = 0.035), and between FeatCNN and GBDT (*p* = 0.033) indicated significant differences. No significant differences were found among the other three models (LR, CNN, and FeatCNN).

For the EEG-driven models, GBDTs also showed superior performance compared to LRs (*p* = 0.0003). However, differences between GBDT and CNN (*p* = 0.13) and between GBDT and FeatCNN (*p* = 0.03) were not statistically significant after correcting for multiple comparisons. No significant differences were observed among the other three models (LR, CNN, and FeatCNN).

To determine the earliest timepoint at which movement onset could be predicted, we trained logistic regression models using a sliding 500 ms window, shifted in 25 ms increments from –2.25 s up to 1.5 s relative to movement onset. This yielded AUC values at each time step, providing a time-resolved measure of the model's ability to distinguish a given period from the resting baseline (see Fig. 4B). We chose LR for this analysis because it is substantially faster to train than more complex classifiers such as GBDTs—an important consideration when fitting a separate model for every 25 ms step across all folds—and it offers straightforward interpretability of feature contributions. In practice, more sensitive classifiers (e.g., GBDTs) may detect movement onset slightly earlier than LR, but likely by only some milliseconds; thus, our LR-based earliest window serves as a conservative benchmark for early prediction of movement.

Statistical analyses revealed that, for LFP-driven models, the first time-window at the AUC was statistically significant above chance (paired *t*-test, *p* < 0.05) was [–0.93 s, –0.43 s], ending at 430 ms before movement initiation. For EEG-driven models, the earliest significant window was [–1.32 s, –0.84 s], ending at 840 ms before movement initiation. We therefore report 430 ms (for LFP) and 840 ms (for EEG) as the earliest timepoints at which movement initiation can be predicted with above-chance accuracy.

We compared the decoding performance of GBDT models trained to detect pre-movement periods ([–500 ms, 0]) with those trained to detect movement execution periods ([0, 500 ms]) using LFP data. Shown in Fig. 4C, for LFP-driven models, the AUC values for movement execution were significantly higher than for pre-movement decoding. A paired *t*-test yielded a *t*-statistic of 14.12 and a *p*-value less than 0.001, with an average AUC increase of 15.3 % when comparing movement execution to pre-movement decoding. For EEG-driven models, the AUC difference was also significant, with a *t*-statistic of 9.02 and a *p*-value below 0.001, and an average relative AUC increase of 14.5 %.

We assessed whether there was a consistent difference in decoding performance when using LFP signals from the thalamus versus EEG signals recorded from the scalp. Paired *t*-tests revealed no significant differences in AUC values between LFP and EEG signals across all models. The uncorrected *p*-values were 0.55 for LR, 0.47 for GBDT, 0.80 for CNN, and 0.87 for FeatCNN. The AUC values for GBDT models using LFP and EEG data are depicted in Fig. 4D. Although fixed-window AUCs did not differ significantly between LFP and EEG, time-resolved analyses indicated earlier detectability with EEG, consistent with distinct temporal profiles of premotor signals across modalities.

### 3.2 Cross-patient variability in modulatory patterns

**Fig. 5:**
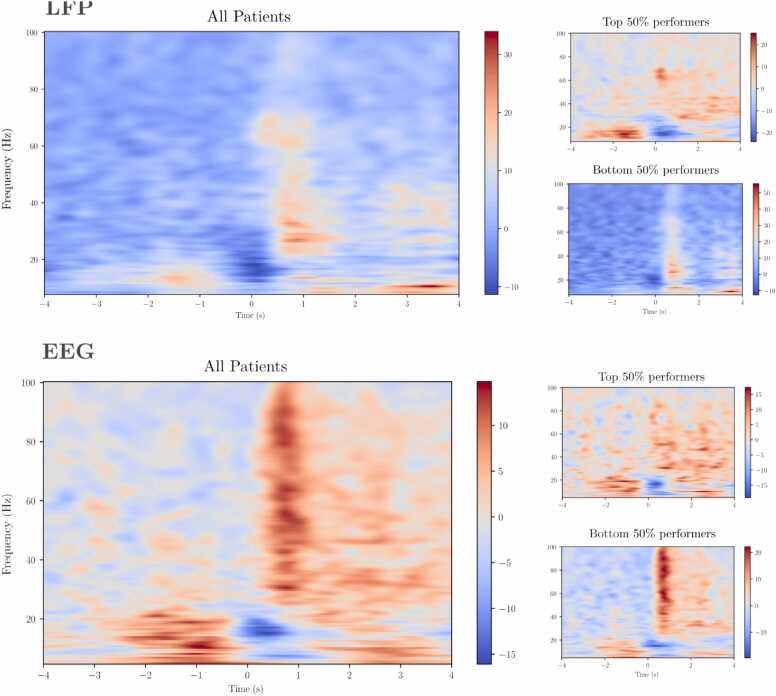
Cohort-level time-frequency decomposition of oscillatory activity time-locked to movement initiation. For each patient, the channel yielding the highest pre-movement decoding accuracy was selected, and the resulting spectrograms were averaged across patients. (Top) LFP signals recorded from the ventral intermediate nucleus (VIM) of the thalamus via externalised DBS leads. (Bottom) EEG signals. (Right) Patients are stratified into two groups based on their individual pre-movement decoding ROC-AUC scores. Upper subplots show time--frequency decompositions for the top-performing half of patients; lower subplots show the bottom half.

Regardless of their architecture, classification models leverage changes in the information content of electrophysiological signals—often manifested as synchronization or desynchronization of activity in canonical frequency bands—to drive decoding performance. To visualise the average modulatory patterns across patients, we computed cohort-level time-frequency decompositions (see Fig. 5). These were calculated using resonator IIR filters with a quality (Q) factor of 20, rectified, and smoothed using a 250 ms kernel. Baseline corrections were applied using values extracted from the rest period ([–4, –2] seconds relative to movement initiation). Additionally, we computed the average time-frequency decompositions separately for the top and bottom 50th percentile performers based on decoding accuracy to explore potential differences in modulatory patterns associated with classification performance.

To investigate the specific modulatory patterns associated with the pre-movement period, we computed modulation indices for individual frequency bands. The modulation index for a specific band was calculated by comparing the power spectral density (PSD) estimates in that band during the pre-movement period with those during the rest period, averaged across trials:

$${\mathrm{MI}}_{\mathrm{band}}=\;\frac{1}{n_{\mathrm{trials}}}\sum_{\mathrm{trial}}\frac{{\mathrm{PSD}}_{\mathrm{pre}-\mathrm{movement}}^{\mathrm{trial},\mathrm{band}}-{\mathrm{PSD}}_{\mathrm{rest}}^{\mathrm{trial},\;\mathrm{band}}}{{\mathrm{PSD}}_{\mathrm{rest}}^{\mathrm{trial},\;\mathrm{band}}}$$

**Fig. 6:**
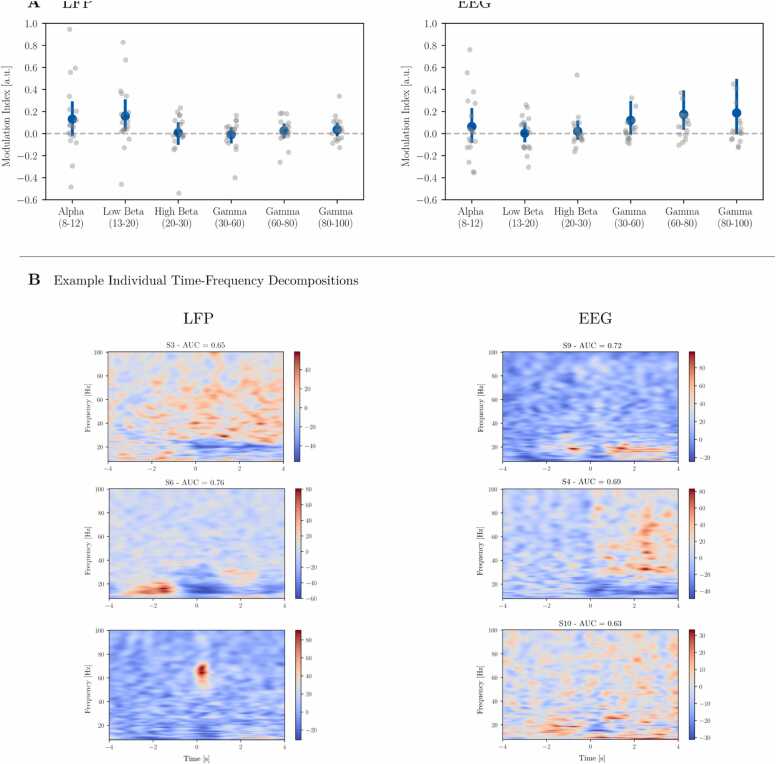
Cross-patient variability in the observed modulatory patterns. (A) Modulation indices for each band. Each dot represents an individual patient, and the blue bars represent the cross-patient mean and 95 % confidence intervals. Negative modulation indices represent desynchronization (decreased activity) during the pre-movement periods, whereas positive indices represent synchronization (increased activity) relative to rest. (B) Individual time-frequency decomposition for a selection of patients, showcasing various modulatory patterns that are present in the dataset.

Modulation indices for each subject and band, along with the mean and 95 % confidence intervals, are depicted in Fig. 6A for both LFP and EEG signals. A negative modulation index corresponds to desynchronization (decreased oscillatory activity) in the respective band during the pre-movement period compared to rest, while a positive modulation index indicates synchronization (increased oscillatory activity). Fig. 6B shows time-frequency decompositions from individual patients, illustrating the variability in modulatory patterns observed across the cohort.

To assess the generalizability of modulatory patterns across patients, we trained logistic regression models under different training conditions and compared their out-of-sample performance. Specifically, we evaluated three training scenarios:

1. **Patient-Specific Training**: Models were trained exclusively on data from the patient of interest.
2. **Combined Training**: Models were trained on data from the patient of interest supplemented with data from other patients.
3. **Cross-Patient Training**: Models were trained exclusively on data from other patients, without including any data from the patient of interest.

**Fig. 7:**
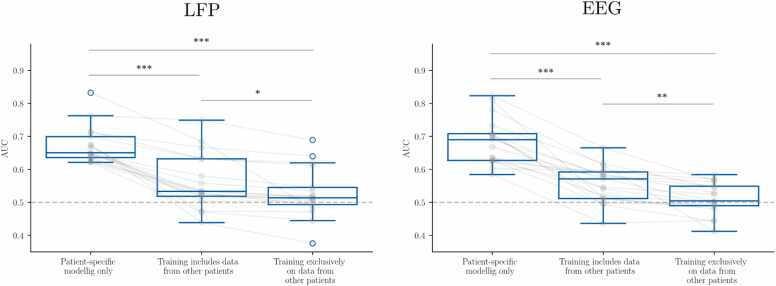
Patient-specific decoding performance under three training schemes: (1) trained solely on the patient’s own data, (2) trained on the patient’s data plus other patients’ data, and (3) trained exclusively on other patients’ data. Including data from other patients during training reduced test-set AUC, demonstrating limited cross-patient generalizability of movement-related modulatory patterns.

We then evaluated the performance of these models on the patient-specific test data. For both LFP and EEG signals, including data from other patients during training led to a degradation in decoding performance compared to patient-specific training (paired *t*-tests, *p* < 0.001 in both cases; see Fig. 7). Furthermore, models trained exclusively on data from other patients performed significantly worse than those trained on combined data (LFP: *p* = 0.011; EEG: *p* = 0.0017). These results suggest that modulatory patterns associated with pre-movement periods exhibit substantial inter-patient variability, limiting the generalizability of models across patients.

### 3.3 Contribution of oscillatory features to decoding

To elucidate the relative contribution of specific bands to decoding performance, we trained LR models using features extracted exclusively from individual canonical bands, as opposed to using all canonical bands simultaneously to drive the regression model. This approach allowed us to quantify the impact of each frequency band on decoding performance, benchmarked against an all-band model.

**Fig. 8:**
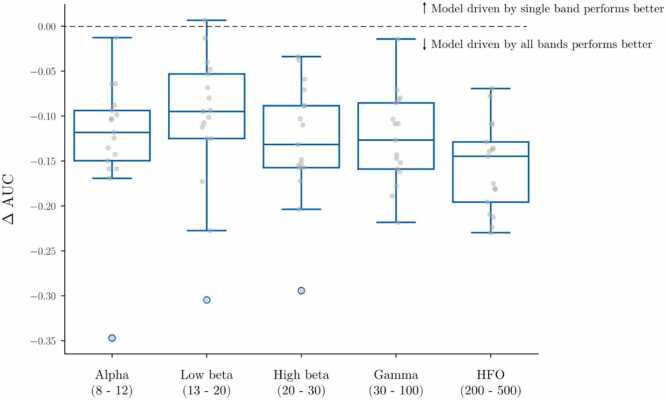
Impact of using individual frequency bands on decoding performance. Distribution plots show the loss in decoding performance (AUC difference) when models are trained using features from a single canonical frequency band compared to a model using all bands. Negative values indicate lower AUC for single-band models relative to the all-band model.

Fig. 8 presents the differences in AUC scores between models trained on individual bands with respect to a model with features from all bands. For every frequency band, relying on only a single band resulted in a degradation in decoding performance. The average decrease in AUC was of 18.8 % ± 8.5 % (mean ± standard deviation) compared to the all-band model. The smallest performance loss was observed in the low beta band (13–20 Hz), with an average AUC decrease of 15.25 % ± 9.9 %. The largest loss occurred in the high-frequency oscillation (HFO) band (200–500 Hz), with an average decrease of 23.01 % ± 6.22 %.

**Fig. 9:**
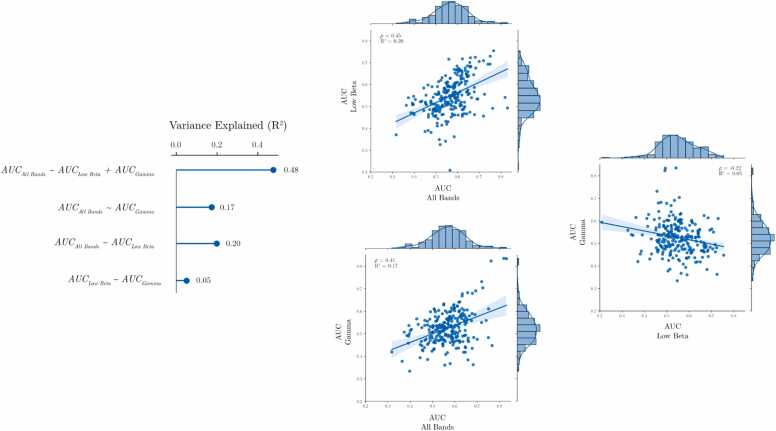
Scatter plots and best-fit linear regression lines showing the relationships between decoding performance (AUC values) of models trained on individual frequency bands and the all-band model. (top) All-band model vs. low beta band model. (bottom) All-band model vs. gamma band model. (right) Low beta band model vs. gamma band model.

To assess whether different frequency bands made independent contributions to decoding performance, we examined how well models trained on individual bands predicted the performance of an all-band model. In practice, we regressed the AUC values from single-band models against the AUC values of the all-band model, as shown in Fig. 9. In this framework, each single-band model’s AUC serves as an independent variable, and the all-band model’s AUC is the dependent variable. If a given frequency band contributes unique information, its standalone AUC should explain a nontrivial portion of the variance in the all-band model’s AUC, even after accounting for other bands.

Our linear regression analyses confirmed that certain bands made statistically significant unique contributions. For example, the AUC from the low-beta (13–20 Hz) model alone explained 20 % of the variance in the all-band model’s AUC (R² = 0.20, p < 0.05), while the gamma (30–100 Hz) model explained 17 % (R² = 0.17, p < 0.05). These coefficients indicate that both low-beta and gamma bands carry information not fully subsumed by the other bands. Notably, the low-beta and gamma AUCs themselves were poorly correlated (R² = 0.05), implying that they capture distinct neural signatures.

To evaluate whether combining these complementary bands improved performance beyond their individual effects, we fit a multiple regression using both low-beta and gamma AUCs as predictors of the all-band AUC. This combined model accounted for 48 % of the variance (R² = 0.48), a substantial increase over either band alone. The significant increase in explained variance when both predictors are included indicates that low-beta and gamma power provide complementary (i.e., nonredundant) information for decoding.

## 4 Discussion

### 4.1 Decoding performance is driven by a spectrum of frequencies

In this study, we investigated the relationship between modulations in canonical frequency bands and the ability of our trained systems to detect pre-movement periods. Our findings suggest that contributions from different frequency bands are both individually relevant as well as relatively independent, indicating that different frequency bands capture distinct neural dynamics associated with pre-movement periods. By leveraging the complementary information from multiple frequency bands, models can achieve superior decoding performance compared to using any single band alone. Our results highlight the importance of incorporating a spectrum of frequency bands in decoding models to capture the complex neural signatures underlying motor preparation and initiation. The additive contributions from different bands support the notion that neural processes involved in movement are distributed across multiple oscillatory activities.

These findings align with previous literature highlighting the importance of multi-band analyses in decoding neural signals. In [39], authors used microelectrodes recordings from the subthalamic nucleus of PD patients to decode a proxy for motor performance. Consistent with the findings shown here, they reported superior decoding performance when models were trained on a broad spectrum of frequencies compared to individual bands. Similarly, [40] and [24] emphasised the simultaneous contributions of multiple frequency bands in subthalamic and thalamic LFPs, as evidenced by the weights assigned by machine learning algorithms to features from various bands. Khawaldeh et al., [41] further demonstrated that including a broad range of frequency bands significantly improved the prediction of clinical movement impairment scores in Parkinson's disease patients when compared to using only the canonical beta band.

For the specific task studied here—the detection of periods leading up to movement initiation—we find that utilising multiple frequency bands simultaneously enhances decoding performance. These results support the multi-band, machine learning-based approach to neural decoding that has been proposed in the literature [29], [42], [43].

Our study also underscores the potential limitations of focusing solely on canonical frequency bands or predefined biomarkers. The variability in modulatory patterns across patients suggests that individualised models, which account for patient-specific neural dynamics across multiple frequency bands, offer better performance. This highlights the importance of data-driven approaches in developing adaptive deep brain stimulation systems and other neurotechnological interventions.

### 4.2 Inference-time computational cost

In the context of aDBS, signal processing pipelines that incorporate machine learning-based components present translationally exciting opportunities [43]. However, the computational environment within which aDBS can be feasibly implemented outside of the clinic remains constrained due to limitations in hardware resources and power consumption [44]. Therefore, efforts to develop algorithms intended for aDBS applications should take these considerations into account to ensure that the systems are practical for real-world deployment.

Estimating the computational complexity of algorithms implemented in high-level scripting languages such as Python or MATLAB is non-trivial, as these implementations may differ significantly from implementations optimised for the embedded systems that would be used in implanted devices. Nonetheless, providing reference values for the computational requirements can offer valuable insights into the feasibility of different algorithms in computationally constrained environments.

To assess the computational demands of our models, we benchmarked the time required to make a single inference-time prediction—that is, to process time-series signals and produce a model output. Benchmarks were performed on an idle server equipped with an AMD EPYC 7402 CPU. Forward passes were run 1000 times, and the median value across those runs was compute and is reported here. This benchmarking provides an approximate measure of the computational efficiency of each algorithm during real-time operation.

The feature extraction step, when using the full feature set, required 187 ms (ms) per prediction. Within this, the estimation of spectral power features—which drive most of the performance of the feature-based models (see 4.2) —took only 5 ms. The time estimates for the forward pass of the different algorithms are outlined in Table 3.

**Table 3:** Benchmarked time [in milliseconds] required for an inference-time prediction.

	LR	GBDT	CNN	FeatCNN
**Full Feature Set**				
Manual Feature Extraction	187	187	N/A	187
Model Inference Time	0.06	0.2	1.0	1.25
**Total Time**	187.06	187.2	**1.0**	188.25
**Spectral Feature Set Only**				
Manual Feature Extraction	5.0	5.0	N/A	5.0
Model Inference Time	0.06	0.2	1.0	1.25
**Total Time**	5.06	5.2	**1.0**	6.25

Abbreviations: LR = Logistic Regression; GBDT = Gradient-Boosted Decision Trees; CNN = Convolutional Neural Network; FeatCNN = CNN with Manually Extracted Features; N/A = Not Applicable.

When using the full feature set, which includes time-domain statistics, autoregressive coefficients, cepstral coefficients, and spectral features, the feature extraction step dominated the computational cost, requiring 187 ms for each prediction. The LR and GBDT models had minimal inference times of 0.06 ms and 0.2 ms, respectively, resulting in total inference times of approximately 187 ms. In contrast, the CNN model, which operates directly on the raw time-series data without manual feature extraction, had a total inference time of 1.0 ms. The FeatCNN model, which combines the CNN with manually extracted features, requires both the feature extraction time (187 ms) and the CNN inference time (1.25 ms), which led to a total inference time of approximately 188 ms.

When using only the spectral feature set, the feature extraction time was significantly reduced to 5 ms. Under this configuration, the total inference times for the LR and GBDT models decreased to approximately 5 ms, whereas the CNN's inference time remained at 1.0 ms, as it does not rely on manual feature extraction. The FeatCNN model's total inference time reduces to approximately 6.25 ms.

### 4.3 Limitations

This study benchmarked the ability to decode upper-limb movements in the milliseconds preceding movement initiation using multiple machine learning algorithms and feature sets. While the findings provide proof-of-feasibility, several limitations must be acknowledged.

#### 4.3.1 Sample size and inter-patient variability

The relatively small cohort (n = 11) limited statistical power and generalisability. Marked heterogeneity in modulatory patterns across patients contributed to variability in decoding performance, and patient-specific models consistently outperformed cross-patient decoders. This heterogeneity likely reflects a combination of factors, including differences in lead placement within VIM/ZI/PSA and individual functional neuroanatomy. While cross-patient decoding is an important translational goal, particularly for reducing calibration burden in adaptive DBS systems, the present dataset was insufficient to support robust feature alignment or domain-adaptation strategies aimed at compensating for inter-patient variability. Mapping patient-specific oscillatory features into a common representational space likely requires larger cohorts, accounting for explicit anatomical localisation of recording contacts, and dedicated transfer-learning or normalisation frameworks. In the absence of these prerequisites, cross-patient models in this study primarily served to illustrate the degree of patient specificity in pre-movement neural signatures, rather than to propose a deployable solution. The limited number of trials per patient, constrained by the acute postoperative recording window (mean ± SD = 67.2 ± 38.8 trials; range: 16–112), restricted the feasibility of learning invariant representations across individuals. Larger, multi-centre datasets will be essential for developing and validating cross-patient decoding schemes that can support generalisable aDBS implementations.

#### 4.3.2 Model interpretability

While we employed established algorithms (logistic regression, gradient-boosted decision trees, convolutional neural networks), model interpretability was limited. The absence of significant correlations between single-band modulation indices and decoding accuracy suggests that non-linear or distributed neural relationships may be driving classification. Future work incorporating explainable machine learning methods and feature attribution techniques could provide deeper insight into the neural mechanisms of movement preparation and initiation.

#### 4.3.3 Real-time validation

All analyses were conducted offline, albeit using a signal processing pipeline compatible with online application (forward-only filtering and causal normalisation). The absence of true real-time testing means we cannot report operational metrics such as sensitivity, specificity, and false-trigger rates in a streaming clinical environment. Such testing—particularly in freely moving patients—remains necessary for assessing clinical feasibility and ensuring safety when developing adaptive DBS schemes.

#### 4.3.4 Multimodal integration

We analysed thalamic LFPs and scalp EEG independently, but our dataset was too limited to reliably characterise the potential benefit of integrating subcortical and cortical signals. Prior work has shown that connectivity within the thalamo-cortical network relates both to volitional movement and to tremor expression. Increased coherence between the thalamus and motor cortex is associated with increased tremor severity [45], [46], and measures of connectivity between these structures are modulated around the onset of volitional movement [47]. These observations support a physiological model in which cortico-cerebellar drive routed through VIM/PSA facilitates movement when appropriately timed, but can also contribute to pathological oscillations within the cerebello-thalamo-cortical loop. Future studies with larger cohorts should test multimodal fusion (e.g., combining cortical EEG with thalamic LFPs) to leverage these cortico-subcortical interactions and potentially enhance both the robustness and lead time of pre-movement decoding.

#### 4.3.5 Task specificity

The experimental tasks used to elicit movements were specific and may not encompass the full range of motor activities encountered in daily life. This could limit the applicability of our models to more naturalistic settings. Expanding the repertoire of tasks and incorporating more ecologically valid movement paradigms could improve the models' relevance and utility in real-world scenarios.

#### 4.3.6 Decoding performance and translational implications

Although decoding performance was statistically above chance for both pre-movement and execution phases, absolute values remain modest in a subset of patients. We do not claim these are sufficient for immediate clinical deployment. Instead, our findings should be interpreted as an early translational step: identifying patient-specific pre-movement neural correlates and demonstrating their feasibility for decoding in a constrained postoperative setting. Moving toward a robust, anticipatory aDBS system will require (i) larger, more diverse datasets collected under naturalistic conditions, (ii) rigorous real-time evaluation, (iii) optimisation of feature extraction and classification to maximise reliability, and (iv) exploration of alternative recording sites, such as motor cortex ECoG, which in some contexts outperforms subcortical LFPs for early movement decoding [27]. By establishing the presence and variability of these early correlates, this study provides an important foundation for future development of early movement-based aDBS strategies for tremor disorders.

### 4.4 Conclusion

We have shown that thalamic LFPs recorded from externalized DBS leads in tremor patients contain reliable pre-movement signals, enabling above-chance decoding of impending upper-limb actions. In our cohort, patient-specific classifiers detected movement onset as early as 430 ms before EMG onset using thalamic LFPs—and 840 ms before EMG onset using scalp EEG. Individualized, patient-specific decoders outperformed cross-patient models, reflecting the inter-subject variability in thalamic oscillatory patterns; this finding confirms that a “one-size-fits-all” approach will likely fail to capture the unique spectral fingerprints of each patient’s tremor network. We also found that no single canonical band suffices: combining features across multiple frequency bands improved decoding accuracy, highlighting that oscillations at different frequencies carry complementary information.

Taken together, our results highlight three contributions: (1) a validation that thalamic LFPs—available from clinically implanted DBS leads—can potentially serve as a real-time, pre-movement biomarker in ET and other tremor disorders; (2) a demonstration that patient-specific, multi-band decoders are needed to accommodate individual differences in recorded thalamic signals; and (3) a benchmarking of classical (logistic regression, GBDTs) versus deep-learning architectures, establishing a baseline for future, more refined algorithms. These insights contribute towards the development of personalized, pre-emptive aDBS systems that initiate stimulation before tremor emerges. More broadly, our work illustrates how invasive LFP sensing and EEG can accelerate the development of adaptive neurostimulation and brain–machine interfaces in clinical practice.

## CRediT authorship contribution statement

**Francesca Morgante:** Writing – review & editing, Resources, Data curation. **Pablo Andrade:** Writing – review & editing, Resources, Data curation. **Michael G. Hart:** Writing – review & editing, Resources, Data curation. **Jamie Brannigan:** Writing – review & editing, Investigation, Formal analysis. **Plazas Fernando Rodriguez:** Writing – review & editing, Writing – original draft, Visualization, Validation, Project administration, Methodology, Investigation, Formal analysis, Data curation, Conceptualization. **Shenghong He:** Writing – review & editing, Supervision, Resources, Project administration, Methodology, Conceptualization. **Huiling Tan:** Writing – review & editing, Validation, Supervision, Resources, Project administration, Methodology, Investigation, Funding acquisition, Conceptualization. **Erlick A. Pereira:** Writing – review & editing, Resources, Data curation. **Veerle Visser-Vandewalle:** Writing – review & editing, Resources, Data curation. **Laura Wehmeyer:** Writing – review & editing, Investigation, Data curation. **Thomas G. Simpson:** Writing – review & editing, Investigation, Data curation. **Rahul S. Shah:** Writing – review & editing, Resources, Data curation.

## Funding

This work was supported by the 10.13039/501100000265Medical Research Council (MC_UU_0003/2, MR/V00655X/1, MR/P012272/1), the Medical and Life Sciences Translational Fund (MLSTF) from the University of Oxford, the National Institute for Health Research (10.13039/100006662NIHR) Oxford Biomedical Research Centre (10.13039/100014461BRC), and the Rosetrees Trust, UK. S.H. was supported by the Guarantors of Brain, the Royal Society Sino-British Fellowship Trust (IES\R3\213123), and Parkinson's UK. R.S.S was personally supported by an NIHR CL award (CL-2021–16–1502) and an RCS England Pump Priming grant funded by Saven Research & Development Fund.

## Declaration of Competing Interest

The authors declare the following financial interests/personal relationships which may be considered as potential competing interests: Shenghong He reports financial support was provided by Guarantors of Brain and The Royal Society. Fernando Rodriguez Plazas and Huiling Tan report financial support was provided by UK Research and Innovation Medical Research Council. Other authors declare that they have no known competing financial interests or personal relationships that could have appeared to influence the work reported in this paper.

## Data Availability

The code required to reproduce the analyses will be shared on the data-sharing platform of the MRC Brain Network Dynamics Unit. The raw data are available upon request by contacting Dr S He.
